# Evaluation of Urinary Tubular Biomarkers in Dogs with Myxomatous Mitral Valve Disease Across ACVIM Stages

**DOI:** 10.3390/vetsci13030243

**Published:** 2026-03-03

**Authors:** Pablo Cardenal-Morales, José Ignacio Cristóbal, Rafael Barrera, Alberto Ezquerra-Durán, Paloma Nicolas, Patricia Ruiz, Ángela Durán-Galea, Francisco Javier Duque

**Affiliations:** 1 MINVET Research Group, Departamento de Medicina Animal, Facultad de Veterinaria, Universidad de Extremadura, 10003 Cáceres, Spain; pablocm@unex.es (P.C.-M.); rabacha@unex.es (R.B.); pnicolash@alumnos.unex.es (P.N.); patriciart@unex.es (P.R.); angeladg@unex.es (Á.D.-G.); javierduque@unex.es (F.J.D.); 2 Veterinary Teaching Hospital, Veterinary Faculty, University of Extremadura, 10003 Cáceres, Spain; 3 Neurogastroenterology and Motility Unit, Gastroenterology Department, Hospital Clinic of Barcelona, 08036 Barcelona, Spain; aezquerra@clinic.cat

**Keywords:** myxomatous mitral valve disease, cardiovascular–renal axis, kidney injury, tubular biomarkers

## Abstract

Myxomatous mitral valve disease (MMVD) is the most common acquired heart valve disease in dogs and it may contribute to cardiovascular–renal axis disorders (CvRD) in dogs. Sensitive and early biomarkers of renal involvement are needed. In this prospective and observational study, 84 dogs were enrolled (20 healthy dogs and 64 dogs with MMVD), categorised using the American College of Veterinary Internal Medicine guidelines. Serum and urinary parameters were analysed, including tubular biomarkers expressed as creatinine-ratios: urinary alkaline phosphatase (uALPc), gamma-glutamyl transferase (uGGTc), N-acetyl-β-D-glucosaminidase (uNAGc), and cystatin C (uCystc). uALPc, uGGTc, and uNAGc were higher in MMVD than in controls; uALPc and uGGTc were increased from stage B1, uNAGc was higher in stages with cardiomegaly (B2 and C+D), and uCystc increased mainly in clinical stages (C+D). Serum renal markers increased only in clinical stages. ROC analysis showed good discrimination for MMVD with uALPc (AUC 0.87) and uGGTc (0.86); for cardiomegaly with uALPc (0.77) and uNAGc (0.75); and for congestive heart failure with SDMA (0.85) and uCystc (0.75). No urinary biomarker was associated with daily furosemide dose. Urinary tubular biomarkers, particularly uALPc and uGGTc, detect early CvRD in dogs with MMVD and complement traditional serum markers.

## Simple Summary

Myxomatous mitral valve disease is the most common heart disease in dogs. Since it is a chronic cardiac disease, it can also affect the kidneys, causing cardiovascular–renal axis disorders. We evaluated whether urine tests could detect early kidney tubular injury in dogs with this disease, for which only a tubular biomarker has been described, urinary neutrophil gelatinase-associated lipocalin. We studied 84 dogs, including 20 healthy controls and 64 dogs with myxomatous mitral valve disease, across different disease stages. We measured four urine tubular biomarkers and compared them with standard serum kidney tests. Urinary alkaline phosphatase and urinary gamma-glutamyl transferase were increased even in early, asymptomatic dogs, urinary N-acetyl-β-D-glucosaminidase was higher in dogs with heart enlargement, and urinary cystatin C and serum kidney biomarkers increased mainly in clinical stages, suggesting that kidney tubular alterations can appear before changes are detected in blood biomarkers. These urine biomarkers were not associated with the diuretic treatment, which could have been a potential cause. Overall, readily available urine tests may help veterinarians detect and monitor early kidney involvement in dogs with myxomatous mitral valve disease within the cardiovascular–renal axis disorders.

## 1. Introduction

Myxomatous mitral valve disease (MMVD) is an acquired cardiac disorder that represents approximately 75% of all heart diseases in dogs and is therefore the most prevalent cardiac disease in this species [1]. It is a slowly progressive condition, most commonly observed in small-breed, elderly dogs (mean age at onset approximately 9.5 years). The most characteristic clinical finding is the detection of a heart murmur on auscultation [2]. Although its aetiology has not been fully determined, a genetic influence is thought to contribute to its development. The clinical presentation in affected dogs is highly variable, ranging from asymptomatic individuals to severe cases with collapse, exercise intolerance, and dyspnoea associated with congestive heart failure (CHF) [3]. Dogs with CHF require chronic treatment, such as daily diuretics [1].

Since it is a chronic cardiac disease [1], affected dogs may develop cardiovascular–renal axis disorders (CvRD) secondary to chronic cardiac disease (CvRD_CH_) [4]. This has been documented as changes in serum and urinary biomarkers of renal damage and function [5,6,7,8,9], which in some cases are already detectable in the preclinical stages of the disease and tend to increase with disease progression [5,8,9].

There are several theories regarding the progression of renal involvement in CvRD_CH_. Some suggest sustained kidney injury due to low cardiac output and renal venous congestion, whereas others propose repeated episodes of acute kidney injury (AKI) over time caused by intermittent cardiac decompensation [10,11]. In addition, the use of diuretics and other drugs as chronic cardiac treatment may contribute to or exacerbate renal damage [4].

For the detection and staging of renal disease, whether acute or chronic, the guidelines of the International Renal Interest Society (IRIS) recommend the use of serum biomarkers such as creatinine (sCr) and symmetric dimethylarginine (SDMA) [12]. However, these biomarkers typically remain within the reference interval until approximately 75% and 40% of nephron mass have been lost, respectively [13,14]. Serum cystatin C (sCyst) has been described as an earlier biomarker of chronic kidney disease (CKD) compared to SDMA and sCr [15,16]. Therefore, the search for more sensitive early biomarkers is a priority [10,16]. New renal biomarkers could facilitate disease monitoring and the management of therapeutic targets to reduce the impact of CvRD [10,17].

In 2015, the consensus statement on CvRD by Pouchelon et al. proposed several potential future urinary biomarkers, some of which have not yet been described in veterinary medicine in this context [4]. These include urinary N-acetyl B-D-glucosaminidase (uNAG), a lysosomal enzyme of tubular cells; urinary gamma-glutamyl transferase (uGGT), a brush-border enzyme of tubular cells; and urinary cystatin C (uCyst), a low molecular weight protein. In addition, other brush-border enzymes, such as urinary alkaline phosphatase (uALP), have been evaluated as renal biomarkers [13]. All of these are urinary biomarkers of tubular damage and/or function, studied mainly in the context of AKI [13,18], with the exception of uCyst, whose use has focused primarily on CKD [18]. To the authors’ knowledge, none of these urinary biomarkers have been studied in MMVD, despite reports of renal tubular damage in this condition [5].

In human medicine, these biomarkers are also used to detect tubular damage [19]. They have been described in the context of cardiovascular–renal syndrome. For example, uGGT and uALP, indexed to urinary creatinine (uCr) as uGGTc and uALPc, have been used in diabetes mellitus for the detection of diabetic nephropathy [20], whereas uCyst has served as a biomarker of renal injury in patients with CHF [21]. Furthermore, uNAG, indexed to uCr as uNAGc, has demonstrated independent prognostic value in patients with heart failure [22], particularly in those with mitral regurgitation [23].

The aim of the present study is to determine uNAG, uGGT, uCyst, and uALP in dogs with stable MMVD (1), to compare them with a control group of healthy dogs (2), and to compare them according to the American College of Veterinary Internal Medicine (ACVIM) classification (3), thereby assessing their potential for the early diagnosis of CvRD_CH_. The main hypothesis is that these biomarkers are altered from the early stages of the disease and that their increase is linked to disease progression.

## 2. Materials and Methods

### 2.1. Animals

This prospective, observational, descriptive, cross-sectional study was performed at the University of Extremadura, Spain. All owners approved and signed an informed consent form. The dogs included were referred, or presented as primary patients, to the cardiology services of the Veterinary Teaching Hospital of the University of Extremadura between February 2023 and October 2025. Because the samples required for this study were obtained during routine health check-ups, the Animal Experimentation Ethics Committee of the University of Extremadura determined that this work could not be considered animal experimentation, in accordance with Royal Decree 53/2013 (Spain).

A total of 84 dogs were enrolled and allocated into two groups: control group (20 healthy dogs) and MMVD group (64 dogs affected by MMVD). The control group consisted of healthy dogs that presented to the hospital for annual check-ups or elective neutering. Inclusion criteria included being older than one year, an unremarkable physical examination, normal blood and urinalysis, and no history of clinical signs or pharmacological treatment during the previous 6 months; antiparasitic treatments and vaccinations were permitted.

Dogs were diagnosed with MMVD according to the following criteria: identification of mitral regurgitation associated with mitral valve thickening or prolapse on echocardiography. All echocardiographic examinations were conducted by an experienced cardiologist. Dogs meeting these requirements were included in the MMVD group and subclassified according to the ACVIM guidelines [1]. Asymptomatic dogs without echocardiographic evidence of cardiomegaly were assigned to the B1 group; asymptomatic dogs with echocardiographic evidence of left-sided cardiac enlargement were assigned to the B2 group; dogs that had experienced clinical signs consistent with congestive heart failure were assigned to the C group; and finally, dogs that required, in addition to standard therapy, a daily dose of ≥8 mg/kg of furosemide for adequate control were assigned to the D group. Patients in the B2 group were enrolled only if they had not received prior treatment. Patients in symptomatic stages (C and D) were included only in the absence of clinical, radiographic or echocardiographic evidence of decompensated congestive heart failure. For statistical purposes, groups C and D were pooled into a single category (C+D group) owing to the limited number of dogs in stage D.

Exclusion criteria included: evidence of urinary pathology or active urinary sediment (>5 erythrocytes/hpf; >5 leukocytes/hpf), presence of sperm in the urine, administration of nephrotoxic drugs within the preceding three months (e.g., nonsteroidal anti-inflammatory drugs), the presence of concomitant systemic diseases such as neoplastic disease, endocrinopathies, infectious disease, or IRIS stage 3 or 4 chronic kidney disease, and the presence of other cardiac conditions (congenital or acquired). Mild pulmonary and aortic insufficiency, as well as tricuspid valve degeneration with or without signs of pulmonary hypertension, were also permitted. Arrhythmias associated with MMVD, as well as their treatment, were allowed. All medications commonly used for MMVD were permitted in the C+D group, including pimobendan, angiotensin-converting enzyme inhibitors (ACEIs), spironolactone and loop diuretics.

Along with the general physical examination, systolic blood pressure (SBP) was measured using a non-invasive ultrasonic Doppler device (Eickemeyer Veterinary Equipment, London, UK), in accordance with the current consensus guidelines for systemic hypertension in dogs [24]. A detailed cardiac auscultation was also performed, and heart murmurs were graded on a six-level scale (I–VI) [3,25].

### 2.2. Clinical Pathology Testing

To minimise the effect of feeding, 5 mL of blood was collected by jugular venipuncture after a 12 h fast. One millilitre was used for haematological determinations (EDTA-K3 tube), and the remaining volume was allowed to clot for one hour in serum tubes; subsequently, the serum was collected for biochemical determinations.

Immediately after collection, haematological analyses were performed using an automated analyser (Element HT5^TM^; Antech Diagnostics, Fountain Valley, CA, USA). The measurements obtained included red blood cell count (RBC), haemoglobin concentration (HGB), haematocrit (HCT), mean corpuscular volume (MCV), mean corpuscular haemoglobin concentration (MCHC), white blood cell count (WBC), differential leukocyte count (neutrophils, eosinophils, lymphocytes, and monocytes), and platelet count.

Serum biochemical measurements were obtained using an automated analyser (Spin200E, Spinreact^®^, Barcelona, Spain) within one hour post serum separation. The parameters measured included glucose, albumin, globulins, sCr, urea, calcium, phosphorus, cholesterol, sodium (Na), potassium (K), chloride (Cl), alkaline phosphatase (ALP), and alanine aminotransferase (ALT). SDMA was determined using a Catalyst Dx Chemistry Analyzer (IDEXX^®^ Laboratories, Inc., Westbrook, ME, USA). Using a latex turbidimetric commercial kit (Cystatin C turbilatex; Spinreact^®^, Barcelona, Spain) sCyst was measured; this technique was previously validated [26] and verified in our laboratory for the commercial assay used [27].

Three millilitres of urine were collected by ultrasound-guided cystocentesis. A urinalysis was performed using reagent strips (Multistix Reagent Strips^®^, Bayer Corporation, Madrid, Spain) and evaluated with an automated reader (Spinreact^®^, Spain). Half a millilitre was reserved for urine culture. The remaining volume was centrifuged at 130× *g* for 5 min, and the sediment was examined microscopically under a 40× objective.

The supernatant was separated for the determination of urine specific gravity using a previously calibrated refractometer (ZUZI 300^®^, Auxilab S.L., Beriáin, Spain). Immediately thereafter, urinary protein concentration was measured using the pyrogallol-molybdate red technique, and creatinine concentration (uCr) was determined using the Jaffé reaction (RAL Diagnostics^®^, SA, Barcelona, Spain), and the urine protein to creatinine ratio (UPC) was calculated. Lastly, uGGT and uALP were determined with an automated biochemical analyser (Spin200E, Spinreact^®^, Barcelona, Spain), following the manufacturer’s recommendations. The GGT assay is based on the enzyme-mediated transfer of a γ-glutamyl group from γ-glutamyl-p-nitroanilide to glycylglycine; the formation rate of 2-nitro-5-aminobenzoic acid is directly proportional to the GGT concentration in the sample; this methodological approach has been previously validated for canine urine in commercial biochemistry analysers [28]. ALP activity was determined by a kinetic colorimetric method using p-nitrophenyl phosphate as substrate (pH 10.4); the photometrically measured formation rate of p-nitrophenol is proportional to ALP activity.

Urinary concentrations of chloride, sodium, and potassium were measured using an automated analyser (MEDICA^®^, Medica Corporation, Bedford, MA, USA) immediately after sample collection. Fractional excretions (FE) of the three electrolytes were subsequently calculated using the formula: FE(x) (%) = (uX × sCr)/(sX × uCr) × 100, where uX represents the urinary concentration of the electrolyte and sX the corresponding serum concentration.

Until the determination of uNAG and uCyst (within one month after collection), the supernatant was stored frozen at −20 °C. uCyst also was determined by a turbidimetric commercial kit (Cystatin C turbilatex; Spinreact^®^, Barcelona, Spain), verified in our laboratory as previously described by Ruiz et al. (2023) [29]. The uNAG was determined by a commercial kit (Diazyme^®^ Laboratories, Poway, CA, USA), previously validated in canine urine [30]. The uNAG assay is based on the enzyme-catalysed hydrolysis of 2-methoxy-4-(2-nitrovinyl)-phenyl-2-acetamido-2-deoxy-β-D-glucopyranoside (MNP-GlcNAc), generating 2-methoxy-4-(2-nitrovinyl)-phenol; after alkalinisation, the chromophore is measured photometrically at 505 nm, and the rate of colour development is proportional to uNAG activity in the sample.

uNAG, uGGT, uCyst, and uALP values were expressed as ratios to uCr: uNAGc, uGGTc, uCystc, and uALPc, respectively.

### 2.3. Statistical Analyses

Normality of continuous variables was assessed with the Shapiro–Wilk test and homogeneity of variances with Levene’s robust test for equality of variances. Depending on the distribution, data are presented as mean ± standard deviation (SD) or median and interquartile range (IQR).

Comparisons between dogs with MMVD and controls were performed using the non-parametric Mann–Whitney U test. Comparisons among MMVD subgroups (B1, B2, and C+D) and the control group were carried out using one-way analysis of variance (ANOVA) for variables with normal distribution and homogeneous variances, or the Kruskal–Wallis test for non-normally distributed variables; when the global test was significant, Bonferroni-adjusted post hoc pairwise comparisons were applied. To assess age as a potential confounder, age-adjusted ordered logistic regression models (ACVIM stage as an ordinal outcome) were fitted, using log-transformed creatinine-indexed urinary tubular biomarkers (uALPc, uGGTc, uCystc, and uNAGc).

Correlations between the variables studied were evaluated using Spearman’s rank correlation coefficient. The association between daily furosemide dose and renal biomarkers (sCr, SDMA, sCyst, uALPc, uGGTc, uNAGc, and uCystc) was assessed by linear regression in dogs receiving furosemide (group C+D). To assess the discriminatory ability of the renal biomarkers, the area under the ROC curve (AUC), sensitivity, specificity, 95% confidence intervals and optimal cut-off values were calculated for three different comparisons: (1) the MMVD group versus the control group, to evaluate their overall diagnostic potential; (2) dogs with cardiomegaly (B2 + C+D) versus dogs without cardiomegaly (B1 + control), to explore their ability to discriminate cardiac remodelling; and (3) clinical versus preclinical stages, to assess their ability to detect the development of CHF. Results were considered significant if *p* < 0.05. Statistical analyses were performed using Stata 17 (StataCorp LLC, College Station, TX, USA).

## 3. Results

Eighty-four dogs were enrolled in the study. Twenty dogs comprised the control group of healthy individuals of various breeds, including fourteen males and six females, of which two males and one female were intact. The remaining 64 dogs constituted the MMVD group, composed of dogs affected by MMVD. This group included 39 males and 25 females of different breeds, with 20 males and 13 females being intact. Dogs in the MMVD group were classified according to the ACVIM consensus [1], into stage B1 (*n* = 21), stage B2 (*n* = 17), and stages C+D (*n* = 26).

The mean age (±SD) of the control group was 3.55 ± 2.24 years, which was significantly lower than that of groups B1 (10.29 ± 2.20 years), B2 (11.21 ± 2.94 years), and C+D (11.13 ± 1.99 years) (*p* < 0.001). The median body weight of the dogs in group C+D (8 kg; IQR: 4.85–16.9) was significantly lower than that of the control group (15 kg; IQR: 8.4–22) and group B2 (8.8 kg; IQR: 5.9–16.3) (*p* < 0.02); no differences were observed compared with group B1 (7.2 kg; IQR: 4.8–15.5).

At enrolment, none of the dogs in groups B1 and B2 were receiving treatment. In group C+D, all dogs (100%) were receiving furosemide, 96.1% pimobendan, and 53.8% both benazepril and spironolactone.

No statistically significant differences in SBP were observed among groups B1, B2, and C+D; median (IQR) values were 150 (140–157.5), 140 (135–160), and 142.5 (132.5–150) mmHg, respectively.

Auscultation revealed a progressive increase in murmur intensity with advancing clinical stage. In group B1, the most frequent murmurs were grade II/VI and III/VI (38.2% and 42.8%, respectively), whereas grade I/VI and IV/VI were less prevalent (14.3% and 4.7%, respectively). In group B2, murmurs of grade III/VI (35.3%), IV/VI (29.4%), and V/VI (29.4%) predominated, with grade VI/VI being less frequent (5.9%). In group C+D, the most prevalent murmurs were grade V/VI (42.3%), followed by grade IV/VI (30.8%), VI/VI (15.4%), and III/VI (11.5%).

### 3.1. Haematology

Haematologic analysis results are shown in Table 1. A significant decrease in RBC was observed in group C+D compared with controls, and HGB and HCT were significantly lower in group C+D than in groups B1 and controls; however, only 11.5% of dogs in group C+D had HGB below the reference range (13.1 g/dL). MCV and MCHC did not differ significantly among groups.

Regarding leukocytes, a trend toward a stress leukogram was noted in clinical stages (C+D). WBC and monocyte counts were significantly higher in group C+D compared with other groups. Neutrophil counts were significantly higher in all MMVD groups compared with controls, with a more marked increase as disease progressed; conversely, lymphocyte counts were significantly reduced in all MMVD groups compared with controls. Finally, eosinophil counts were significantly lower in group C+D compared with controls, and platelet counts were significantly higher in groups B2, and C+D compared with controls.

### 3.2. Serum Biochemical Parameters

Serum biochemistry results are shown in Table 2. Glucose concentrations were significantly higher in all MMVD subgroups compared with controls. Total protein levels were higher in all MMVD subgroups, reaching statistical significance in groups B1 and C+D; this was mainly due to an increase in globulins, which was significant only in group C+D. Albumin, calcium, cholesterol, sodium, and ALT did not differ significantly among groups.

Serum phosphorus was significantly higher in the control and C+D groups than in groups B1 and B2. Regarding electrolytes, chloride was significantly higher in group B2, and potassium was significantly lower in all diseased groups compared with controls, resulting in a significantly higher Na/K ratio in MMVD groups. Alkaline phosphatase was significantly higher in stage B2 compared with controls.

Markers of renal function showed that urea and SDMA were significantly increased in group C+D compared with other groups; moreover, SDMA in group B1 was significantly lower than in controls. Serum creatinine was significantly higher in group C+D compared with group B1, and sCyst was significantly higher in group C+D than in controls.

### 3.3. Urinalysis

Urinalysis results are presented in Table 3 and Table 4. Urine pH did not differ significantly among groups. Urine specific gravity decreased progressively across stages, reaching statistical significance from stage B2 onward; group C+D showed the lowest value compared with the other groups. UPC was significantly increased in all MMVD subgroups. Urinary creatinine was significantly lower in group C+D than in other groups.

**Figure 1:**
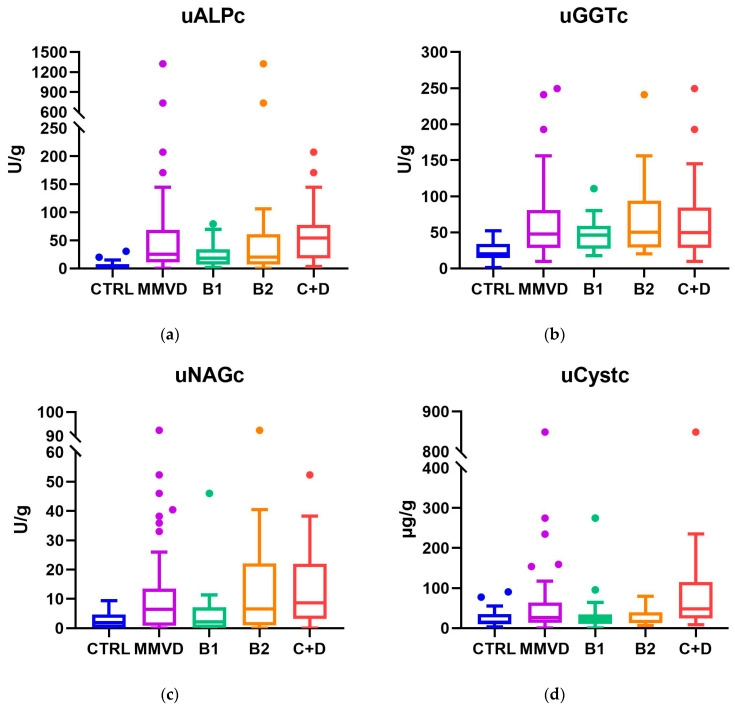
(**a**) uALPc, (**b**) uGGTc, (**c**) uNAGc, (**d**) uCystc, of healthy dogs (CTRL), dogs with myxomatous mitral valve disease (MMVD), and its ACVIM subgroups (B1, B2, and C+D). Data are presented as boxes and whiskers. Each box includes the interquartile range, the line within a box represents the median and the whiskers represent values within 1.5×interquartile range. Outliers are depicted by circles.

Tubular biomarkers expressed as a ratio to uCr showed a progressive increase across disease stages for uGGTc and uALPc, with uALPc being significantly higher in clinical stages compared with B1. uNAGc was significantly higher in groups C+D and B2 than in controls and B1, whereas uCystc showed a significant increase only in clinical stages compared with other groups (Figure 1). In age-adjusted models, age was associated with a higher clinical stage. After adjustment for age, all four creatinine-indexed urinary tubular biomarkers remained independently associated with clinical stage (Supplementary Table S1).

When tubular biomarkers were compared between healthy dogs and the MMVD group, uGGTc, uALPc, and uNAGc were significantly higher in the MMVD group, whereas no significant difference was found for uCystc (Figure 1). The absolute values of the tubular biomarkers across groups are reported in Supplementary Table S2.

Fractional excretions of electrolytes revealed that FE Cl was significantly higher in group C+D than in B1; FE Na was significantly higher in group C+D than in controls and B1; and FE K was significantly higher in group C+D than in all other groups.

### 3.4. Receiver Operating Characteristic Curves

Regarding the ROC curves calculated, for MMVD vs. controls, the biomarkers with the highest AUCs were uALPc (AUC = 0.87), uGGTc (AUC = 0.86), and uNAGc (AUC = 0.69). For the presence of cardiomegaly, uALPc (AUC = 0.77), uNAGc (AUC = 0.75), and uGGTc (AUC = 0.74) were the most informative. For clinical vs. preclinical stages, SDMA (AUC = 0.85), sCr (AUC = 0.76), and uCystc (AUC = 0.75) showed the best discriminative ability. Detailed AUC values, sensitivity, specificity, and cut-off points are presented in Table 5.

**Figure 2:**
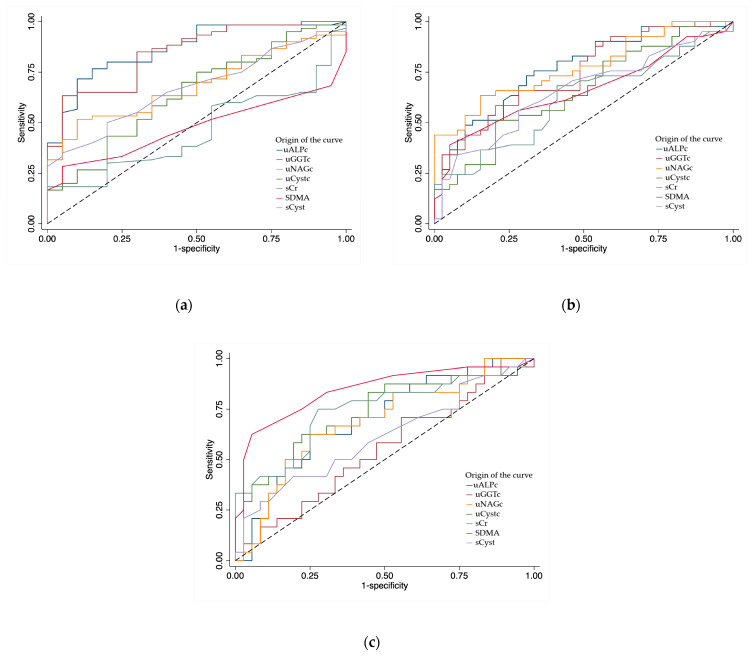
ROC curves comparing the discriminatory ability of uALPc, uGGTc, uNAGc, uCystc, sCr, SDMA, and sCyst in detecting (**a**) myxomatous mitral valve disease, (**b**) cardiomegaly, and (**c**) chronic congestive heart failure. Abbreviations: uALPc—urinary alkaline phosphatase/creatinine ratio; uGGTc—urinary gamma-glutamyl transferase/creatinine ratio; uNAGc—urinary N-acetyl-β-D-glucosaminidase/creatinine ratio; uCystc—urinary cystatin C/creatinine ratio; sCr—serum creatinine; SDMA—symmetric dimethylarginine; sCyst—serum cystatin C.

Overall, these findings indicate that urinary tubular biomarkers, particularly uALPc and uGGTc, provide superior discriminatory performance for MMVD detection and progression compared with traditional serum markers (Figure 2).

### 3.5. Correlation and Regression Analyses

No significant association was detected between any renal biomarkers (sCr, SDMA, sCyst, uALPc, uGGTc, uNAGc, and uCystc) and the daily furosemide dose (R^2^ = 0.07, *p* = 0.188; R^2^ = 0.03, *p* = 0.39; R^2^ = 0.11, *p* = 0.10; R^2^ = 0.02, *p* = 0.517; R^2^ = 0.001, *p* = 0.871; R^2^ = 0.06 *p* = 0.99; and R^2^ = 0.00, *p* = 0.214, respectively).

No significant correlations were observed between serum renal biomarkers and urinary tubular biomarkers. Within the tubular biomarkers, a strong positive correlation was found between uALPc and uGGTc (r = 0.673; *p* < 0.01). Moderate positive correlations were identified between uNAGc and uGGTc (r = 0.312; *p* < 0.01) and uNAGc and uCystc (r = 0.305; *p* < 0.01). Weak positive correlations were found between uNAGc and uALPc (r = 0.284; *p* < 0.05) and uALPc and uCystc (r = 0.225; *p* < 0.05).

UPC exhibited a moderate positive correlation with uGGTc (r = 0.546; *p* < 0.01) and uALPc (r = 0.496; *p* < 0.01). Finally, a moderate negative correlation was observed between HGB and sCyst (r = −0.312; *p* < 0.01) and a weak negative correlation between HGB and uCystc (r = −0.290; *p* < 0.01) (Table 6).

## 4. Discussion

The present study aimed to evaluate urinary tubular biomarkers (uGGT, uALP, uCyst and uNAG) in clinically stable dogs with MMVD to determine their potential as early indicators of CvRD_CH_ and to assess the prevalence of tubular alterations across different disease stages.

CvRD, analogous to cardiorenal syndrome in human medicine [31], describes the disruption of heart–kidney interplay leading to renal and/or cardiovascular dysfunction [4]. Within this framework, MMVD may contribute to CvRD as a chronic cardiac disease that can secondarily affect renal function [32]. In dogs with MMVD, CKD prevalence increases with disease progression [33,34], and MMVD has been identified as a risk factor for CKD progression in dogs affected by both conditions [35].

While CKD is a negative prognostic factor in human mitral regurgitation [23], evidence in dogs with MMVD is inconsistent: some studies have associated increases in urea, sCr and sCyst with worse outcomes [36,37], whereas others reported no survival impact of azotaemia after CHF onset [38,39] and no differences in sCr between dogs that survived or died within 6 months [40]. Overall, current data do not support firm conclusions regarding the prognostic value of conventional renal biomarkers in canine MMVD.

Anaemia can contribute to CvRD by promoting tissue hypoxia and subsequent renal and cardiac injury [32]. In human medicine, cardiorenal anaemia syndrome highlights its clinical relevance [41]. While anaemia is common in dogs with CKD [42], it is not consistently reported in MMVD [43,44]. In the present study, anaemia (HGB < 13.1 g/dL) was uncommon (0%, 11%, and 11.5% in groups B1, B2, and C+D, respectively), but HGB, RBC, and HCT were significantly lower in clinical stages, suggesting a mild tendency toward anaemia. Although anaemia has been associated with advanced MMVD and higher sCr [43], moderate and weak negative correlation between HGB and sCyst and uCystc, respectively.

Regarding leukocyte profiles, our results were consistent with a stress leukogram that was more pronounced in clinical stages [9,45,46]. Higher WBC counts have been associated with poorer 6-month outcome [40], supporting the presence of systemic inflammatory alterations in dogs with MMVD [47].

Assessment of renal function in MMVD has relied on blood parameters such as sCr, SDMA, and sCyst, which reflect changes in functional renal mass and indirectly estimate glomerular filtration rate (GFR) [14,26,48]. These markers typically increase in the clinical stages [5,7,8,9,34,49], consistent with our findings for serum sCr, SDMA, and sCyst suggesting reduced GFR in advanced disease. Given patients’ age, some changes may reflect pre-existing CKD, whose prevalence rises with age [50]. However, subgroup ages did not differ significantly, and CKD is more prevalent in dogs with MMVD than in those without cardiac disease [34], suggesting the changes are more likely attributable to MMVD and CvRD than age alone.

Urinalysis revealed a significant increase in UPC across all MMVD stages, consistent with reports in advanced stages [7]. However, no stage-dependent differences were observed, contrasting with earlier findings [5]. Only 15% of MMVD dogs were proteinuric (UPC > 0.5), which may relate to patient age, as similar proteinuria prevalence has been documented in apparently healthy geriatric dogs [51]. Importantly, proteinuria did not worsen with disease progression in our cohort.

FE Na, FE K, and FE Cl increased significantly in clinical stages, consistent with furosemide inhibition of the Na^+^-K^+^-2Cl^−^ cotransporter in the loop of Henle, reducing electrolyte reabsorption and enhancing urinary excretion [52]. FE values are influenced by the interval between furosemide administration and sample collection. A recent study demonstrated higher FE Na, FE Cl, and FE K shortly after drug administration compared with later intervals, with morning samples showing the highest values [53]. As our patients were evaluated in the morning, our FE values match those reported for the morning group in that study.

Few studies have assessed urinary biomarkers in MMVD, and only one tubular damage biomarker, urinary neutrophil gelatinase-associated lipocalin (uNGAL), has been reported [5]. uNGAL is a protein normally filtered by glomerulus and reabsorbed by the tubule, but proximal or distal tubular injury increases its synthesis and secretion, elevating urinary concentrations [54]. Additional CvRD-related biomarkers should be investigated to improve understanding of tubular pathophysiology in MMVD [4].

uGGT and uALP are brush-border enzymes released into urine after loss of cytoplasmic membrane integrity, indicating proximal tubular injury [55]. Both biomarkers can be measured using standard serum/plasma techniques, making them inexpensive and readily applicable in clinical practice [54]. uNAG is a lysosomal enzyme present in proximal tubular cells normally excreted via lysosomal fusion with the cell membrane. Increased urinary NAG may reflect lysosomal system activation [56] or tubular damage leading to lysosomal release [55], and it is a mixed-type marker. Proximal tubular cells are metabolically very active and prone to early damage [57]. In the absence of tubular injury, urinary concentrations of these enzymes remain very low [55].

Increases in these three biomarkers are mainly reported in AKI [13,18,58] and they have been correlated with proximal tubular histopathological changes in bitches with pyometra confirmed by renal biopsy [59]. uNAGc and uGGTc have also been described in CKD, with early elevations in canine leishmaniosis [29,60]. However, Nivy et al. did not detect differences in uGGTc and uALPc between healthy dogs and those with CKD [61], and Smets et al. reported substantial overlap in uNAGc values between dogs with and without CKD [62]. This may reflect the fact that not all CKD cases exhibit persistent tubular involvement over time. Following an insult that leads to CKD, once tubular injury subsides, these biomarkers may return to baseline [63].

In canine MMVD, tubular damage is present from the earliest stages of disease, as demonstrated by increases in uNGAL normalised to urinary creatinine (uNGALc) [5]. This aligns with our uGGTc and uALPc findings, which showed elevations from stage B1 and exhibited good discriminatory ability between dogs with MMVD and healthy controls, based on ROC curve performance. Furthermore, a strong positive correlation was observed between uGGTc and uALPc, consistent with their shared localisation in tubular cells [55] and similar interpretation of their increase. Notably, uALPc not only increased in early stages but also demonstrated a significant additional rise in advanced stages compared with initial stages, indicating disease progression, similar to uNGALc [5]. The moderate positive correlations between UPC and both uALPc and uGGTc are consistent with previous findings on uNGALc in dogs with MMVD [5] and may relate to low-grade proteinuria secondary to impaired proximal tubular reabsorption [64]. It is unlikely that uALP and uGGT originate from inappropriate passage across the glomerular barrier, given their large molecular size [55].

Conversely, uNAGc was significantly increased in stages associated with cardiomegaly, a distinction not observed for the other biomarkers in this study nor for uNGALc in Troia et al. [5]. This is supported by its moderate ability to discriminate dogs with cardiomegaly from those without. These findings suggest tubular injury in stage B1 is less severe than in B2, as lysosomal enzymes (uNAG) indicate more pronounced damage than brush-border enzymes (uGGT and uALP) due to their intracellular localisation [64]. This supports the presence of disease progression, with tubular damage in stage B1 and both tubular damage and dysfunction in stages B2, C, and D, becoming progressively more pronounced across stages. Additionally, uNAGc correlated with all three others, reinforcing its role in tubular damage and dysfunction [55,56].

In humans, increases in uNAGc and uNGALc have been reported in stable chronic systolic heart failure. Unlike uNGALc, uNAGc provided prognostic information independent of GFR, underscoring the relevance of tubular injury itself in this population [22]. More recently, uNAGc was found to correlate with CHF stage, outcome, HGB, and echocardiographic parameters in patients with CHF and mitral regurgitation [23], supporting uNAGc as an important biomarker in cardiorenal syndrome. In contrast, we did not observe a correlation between uNAGc and haemoglobin in our study.

The observed increases in urinary biomarkers without concurrent changes in serum markers during early disease stages highlight their superior ability to detect early renal alterations [54]. In human AKI, tubular injury may occur without sufficient damage to alter GFR [65]. This is relevant in CvRD, where renal impairment may result from intermittent cardiac decompensations causing repeated renal insults or from sustained renal injury driven by persistent cardiac dysfunction [10].

Cystatin C is a protein synthesised at a constant rate by most nucleated cells. It freely passes through the glomerular barrier and is reabsorbed and metabolised by tubular cells. Thus, its urine increase has been associated with tubular dysfunction [66]. It has been studied mainly in CKD, with increases from early stages of canine leishmaniosis [29] and proposed as an early CKD indicator, even in non-azotemic stages [67]. In our study, uCystc showed significant elevation only in clinical stages, possibly reflecting an incipient reduction in GFR already detected by serum markers. This is supported by its moderate ability to discriminate MMVD dogs with stable CHF from those without it. Human patients with acute heart failure and higher proteinuria exhibited greater mortality and higher uCyst concentrations, suggesting an indirect association between uCyst elevation and worse prognosis, likely reflecting more advanced renal dysfunction within the cardiorenal context [68].

Considering optimal cut-off points (Table 5), serum markers showed minimal variation across the three successive stages of disease progression (MMVD, cardiomegaly, and CHF), with SDMA remaining unchanged. In contrast, urinary biomarkers exhibited a clear upward trend in cut-off values, indicating progressive tubular alteration as disease advances. Overall AUC values were equal to or higher for urinary than serum biomarkers, and they performed better in the preclinical stages, consistent with previous evidence that tubular biomarkers outperform serum biomarkers for early kidney injury detection [16,54]. Serum biomarkers correlated among themselves, as tubular biomarkers did, but no correlations were found between serum and tubular markers, underscoring their distinct clinical significance.

Several mechanisms may contribute to tubular alteration described in CvRD_CH_. First, hemodynamic factors such as venous congestion and renal hypoperfusion have been implicated [4,31,69]. Venous congestion, assessed indirectly by echocardiography in human and veterinary medicine, has been associated with increased tubular biomarkers in patients with cardiac disease [23,70]. However, in the present study, no differences in SBP were observed between groups, which contrasts with previous findings reporting significant SBP reductions in advanced stages [71], suggesting renal hypoperfusion as a potential contributor. Although human studies have not demonstrated a correlation between tubular biomarkers and SBP, they have shown associations with reduced ejection fraction [23], suggesting renal hypoperfusion secondary to episodic, subclinical cardiac events may occur intermittently [10]. Neurohormonal activation, particularly involving the renin–angiotensin–aldosterone system (RAAS) and the sympathetic nervous system, is considered a key mechanism in MMVD [4,31,69]. Accordingly, current guidelines recommend RAAS-modulating therapy in clinical stages, although the supporting evidence is limited [1]. Finally, oxidative stress and inflammation should also be considered [4,31,69], the latter being consistent with our leukogram findings. These mechanisms may be present from the early stages and progress alongside disease severity [10].

Another factor described in CvRD_CH_ is the administration of diuretics during clinical stages, which may induce hypovolemia and stimulate the RAAS, potentially causing or exacerbating renal injury [4,31]. However, in the present study, none of the urinary biomarkers showed a significant association with the daily furosemide dose, suggesting that diuretic therapy is unlikely to play a primary role in CvRD_CH_, consistent with previous reports on uNGALc [5]. Interestingly, human studies have reported that withdrawal of diuretic therapy in patients with CHF led to increases in uNAG, which normalised upon reintroduction of treatment [72]. These observations further support that the biomarker elevations observed in clinical stages are more likely attributable to the aforementioned pathophysiological mechanisms rather than diuretic use.

The present study has several limitations. The control group was not matched for age and body weight with the dogs with MMVD. Because stage D MMVD has low prevalence and poor life expectancy, only a small number of dogs were included and were pooled with stage C, preventing us from determining whether these subgroups behave similarly. The C+D group received non-homogenous treatments. Another constraint is the lack of blinding regarding clinical assessments and laboratory analyses. The uALP assay has not been analytically validated for canine urine; therefore, uALP results should be interpreted with caution. In addition, urinary creatinine was determined using the Jaffé reaction, which is less sensitive than enzymatic creatinine assays; this should be considered when interpreting creatinine-indexed urinary biomarker ratios. Knowledge of the dogs’ group allocation (control or MMVD) may have biased the findings. Finally, despite evaluating several tubular biomarkers, no complementary tests such as urinary protein electrophoresis were performed, which would have allowed us to determine whether the proteinuria reported had a tubular, glomerular, or mixed origin. Likewise, GFR was not directly measured and renal biopsies were not performed to accurately characterise renal status; mainly due to the invasive nature of these procedures.

## 5. Conclusions

In conclusion, dogs with MMVD develop CvRD_CH_ from early stages, and these disorders become more pronounced as the disease progresses, as evidenced by increases in tubular urinary biomarkers (uNAGc, uCystc, uALPc, and uGGTc), the last two of which are accessible to most clinicians. This increase is not associated with the use of diuretics, underscoring the importance of other pathophysiological connections between the kidney and the cardiovascular system within CvRD. Future investigation of the progression of these alterations and their impact on prognosis will be key to understanding their clinical relevance.

## Figures and Tables

**Table 1:** Haematology results in healthy dogs and dogs with MMVD divided by ACVIM.

	Control Group	B1 Group	B2 Group	C+D Group
**RBC (×10^6^/µL)**	6.87 (6.48–7.21) π	6.86 (6.37–7.24)	6.49 (5.78–6.81)	6.16 (5.65–7.01) π
**HGB (g/dL)**	17 (16.5–18.05) π	16.6 (15.8–18.6) /	16.2 (14.3–17)	14.6 (14–16.6) π/
**HCT (%)**	46 (43.95–48) π	45.8 (43.5–49.4) /	43.4 (38.1–46.3)	40.15 (37.4–45.4) π/
**MCV (fL)**	67.87 ± 2.26	68.15 ± 3.0	67.71 ± 2.64	67.77 ± 2.46
**MCHC (g/dL)**	36.87 ± 2.26	36.34 ± 1.21	36.22 ± 1.6	36.49 ± 1.34
**WBC (×10^3^/μL)**	7.72 (6.72–9.11) π	6.87 (6.18–8.03) /	8.2 (7.06–11.96) -	13.32 (9.52–18.55) π/-
**Neutrophil count (×10^3^/μL)**	4.55 (3.11–5.72) π&	4.94 (4.18–5.66) /	5.51 (4.79–7.15) &-	9.9 (6.81–15.16) π/-
**Eosinophil count (×10^3^/μL)**	0.385 (0.28–0.52) π	0.21 (0.18–0.34)	0.25 (0.13–0.31)	0.235 (0.07–0.35) π
**Lymphocyte count (×10^3^/μL)**	2.22 (2.02–2.93) *&π	1.32 (1.08–1.81) *	1.4 (1.15–1.95) &	1.59 (1.04–2) π
**Monocyte count (×10^3^/μL)**	0.51 (0.41–0.59) π	0.4 (0.36–0.55) /	0.54 (0.38–0.75) -	1.09 (0.83–1.39) π/-
**Platelet count (×10^3^/μL)**	260 (183.5–304) &π	298 (231–381)	307 (273–408) &	370 (318–415) π

Abbreviations: RBC—red blood cells; HGB—haemoglobin concentration; HCT—haematocrit; MCV—mean corpuscular volume; MCHC—mean corpuscular haemoglobin concentration; WBC—white blood cells. Values are presented as mean ± SD or median (IQR). * *p* < 0.05 indicates a statistically significant difference between control group and B1 group; & between control group and B2 group; π between control group and C+D group; / between B1 group and C+D group; - between B2 group and C+D group.

**Table 2:** Serum biochemical results in healthy dogs and dogs with MMVD divided by ACVIM.

	Control Group	B1 Group	B2 Group	C+D Group
**Glucose (mg/dL)**	88.95 (81.51–92.37) *&π	102.96 (95.54–118.5) *	98.39 (93.12–104) &	107.295 (94.14–116) π
**Total proteins (g/dL)**	6.11 ± 0.3 *π	6.64 ± 0.57 *	6.58 ± 0.53	6.57 ± 0.62 π
**Albumin (g/dL)**	3.26 ± 0.15	3.41 ± 0.3	3.41 ± 0.26	3.24 ± 0.28
**Globulins (g/dL)**	2.86 ± 0.28 π	3.22 ± 0.5	3.17 ± 0.45	3.32 ± 0.54 π
**Calcium (mg/dL)**	10.16 (9.83–10.45)	10.65 (10.09–11.8)	10.75 (10.36–10.97)	10.37 (9.69–11.37)
**Phosphorus (mg/dL)**	4.13 (3.81–4.68) *&	3.3 (2.72–3.71) */	3.3 (2.47–3.72) &-	3.91 (3.34–5.29) /-
**Cholesterol (mg/dL)**	218.5 (185.5–265.5)	234 (183–287)	233 (175–272)	236 (204–256)
**Sodium (mEq/L)**	149.25 (145–150.2)	150 (147–153.7)	147.4 (146.2–157)	146 (145–152.8)
**Potassium (mEq/L)**	5.21 (4.8–5.35) *&π	4.41 (3.89–4.75) *	4.34 (4–4.68) &	4.205 (3.82–4.71) π
**Chloride (mEq/L)**	114.35 (108.55–122.9) &	119 (113.2–121.8)	123 (118.5–125.3) &	118.55 (115.9–121.9)
**Na/K**	28.75 (27.81–30.86) *&π	33.18 (30.96–38.43) *	34.57 (29.57–36.85) &	34.99 (32.17–37.47) π
**ALP (U/L)**	88 (56.5–138.5) &	128 (69–320)	167 (130–367) &	142 (73–331)
**ALT (U/L)**	37.5 (32–52)	49 (38–65)	54 (45–93)	40.5 (30–78)
**Creatinine (mg/dL)**	1.12 (1.01–1.2)	0.98 (0.91–1.09) /	1.08 (0.98–1.16)	1.2 (1.1–1.94) /
**Urea (mg/dL)**	38.7 (33.05–44.95) π	35.6 (28.7–41.7) /	35.5 (26.7–43) -	65.9 (50.2–93) π/-
**SDMA (μg/dL)**	10 (9–11.5) *π	8 (7–10) */	9 (8–11) -	13 (11–17) π/-
**Cystatin C (mg/L)**	0.15 ± 0.04 π	0.17 ± 0.07	0.18 ± 0.06	0.21 ± 0.09 π

Abbreviations: ALT—alanine aminotransferase; ALP—alkaline phosphatase; SDMA—symmetric dimethylarginine; Na/K—sodium/potassium ratio. Values are presented as mean ± SD or median (IQR). * *p* < 0.05 indicates a statistically significant difference between control group and B1 group; & between control group and B2 group; π between control group and C+D group; / between B1 group and C+D group; - between B2 group and C+D group.

**Table 3:** Urinalysis results in healthy dogs, dogs with MMVD, and dogs with MMVD divided by ACVIM.

	Control Group	B1 Group	B2 Group	C+D Group
**pH**	6.8 ± 0.83	7.14 ± 1	7.44 ± 0.86	7.11 ± 0.81
**USG**	1.043 (1.029–1.05) π&	1.031 (1.023–1.041) /	1.031 (1.021–1.037) &-	1.013 (1.011–1.016) π/-
**UPC**	0.05 (0.01–0.14) *&π	0.17 (0.11–0.4) *	0.21 (0.12–0.47) &	0.19 (0.13–0.29) π
**Urinary creatinine (mg/dL)**	168.89 (115.49–222.1) π	125.48 (94–218.3) /	151.16 (83.35–199.65) -	40.76 (29.68–75.73) π/-
**FE Na (%)**	0.34 (0.23–0.7) π	0.66 (0.3–0.77) /	0.74 (0.28–1.17)	1.37 (0.6–3.48) π/
**FE Cl (%)**	0.89 (0.56–1.17)	0.64 (0.32–0.95) /	0.8 (0.48–1.69)	1.52 (0.66–3.69) /
**FE K (%)**	14.51 (9.74–17.52) π	16.5 (10.51–22.89) *	14.67 (10.56–24.22) &	28.28 (17.17–52.96) *&π

Abbreviations: UPC—urinary ratio proteins/creatinine; USG—urine specific gravity; FE Na—fractional excretion of sodium; FE Cl—fractional excretion of chloride; FE K—fractional excretion of potassium. Values are presented as mean ± SD or median (IQR). * *p* < 0.05 indicates a statistically significant difference between control group and B1 group; & between control group and B2 group; π between control group and C+D group; / between B1 group and C+D group; - between B2 group and C+D group.

**Table 4:** Tubular biomarkers result in healthy dogs, dogs with MMVD, and dogs with MMVD divided by ACVIM.

	Control Group	MMVD Group	B1 Group	B2 Group	C+D Group
**uALPc (U/g)**	2.56 (0.44–7.935) ^*&π	25.46 (11.61–68.59) ^	18.27 (7.55–32.04) */	20 (7.23–54.81) &	53.93 (17.68–77.04) π/
**uGGTc (U/g)**	20.22 (14.86–34.01) ^*&π	47.73 (28.52–80.47) ^	46.15 (27.80–53.24) *	50 (29.12–93.47) &	49.83 (28.7–81.49) π
**uCystc (µg/g)**	15.06 (10.12–34.63) π	26.89 (13.74–63.83)	22.56 (10.48–33.53)/	16.75 (13.23–37.78) -	48.09 (24.53–114.32) π/-
**uNAGc (U/g)**	1.89 (0.107–4.63) ^&π	6.42 (0.91–13.51) ^	2.17 (0.08–6.77) !/	6.56 (0.94–19.65) &!	8.59 (3.38–21.38) π/

Abbreviations: uALPc—urinary ratio alkaline phosphatase/creatinine; uGGTc—urinary ratio gamma-glutamyl transferase/creatinine; uCystc—urinary ratio cystatin C/creatinine; uNAGc—urinary ratio N-acetyl B-D-glucosaminidase/creatinine. Values are presented as mean ± SD or median (IQR). * *p* < 0.05 indicates a statistically significant difference between control group and B1 group; & between control group and B2 group; π between control group and C+D group; ! between B1 group and B2 group; / between B1 group and C+D group; - between B2 group and C+D group; ^ between control group and MMVD group.

**Table 5:** The cut-off values, sensitivity, and specificity of each parameter for the prediction of MMVD, cardiomegaly, and chronic congestive heart failure based on ROC curve analysis.

	sCr (mg/dL)	SDMA (μg/dL)	sCyst (mg/L)	uALPc (U/g)	uGGTc (U/g)	uNAGc (U/g)	uCystc (µg/g)
	**MMVD vs. Control**						
**AUC**	0.46	0.48	0.66	0.87	0.86	0.69	0.64
**95% CI**	0.33–0.6	0.36–0.6	0.54–0.79	0.78–0.95	0.77–0.94	0.57–0.8	0.51–0.78
**Cut-offs**	1.06	11	0.16	9.29	26.17	5.38	24.53
**Sensitivity (%)**	59.4	43.5	63.5	77.4	85.9	54	58.7
**Specificity (%)**	45	60	60	85	70	85	65
	**B1 + Control vs. B2 + C+D (Cardiomegaly)**						
**AUC**	0.63	0.67	0.65	0.77	0.74	0.75	0.67
**95% CI**	0.51–0.75	0.55–0.79	0.63–0.77	0.66–0.87	0.64–0.85	0.65–0.86	0.55–0.78
**Cut-offs**	1.08	11	0.17	17	46.98	6.41	33.28
**Sensitivity (%)**	69.8	57.1	60.5	73.8	60.5	62.8	55.8
**Specificity (%)**	58.5	72.5	65	67.5	78	82.5	72.5
	**B1 + B2 vs. C+D (Chronic congestive heart failure)**						
**AUC**	0.76	0.85	0.6	0.71	0.56	0.66	0.75
**95% CI**	0.63–0.88	0.75–0.95	0.45–0.75	0.57–0.84	0.42–0.71	0.52–0.8	0.62–0.87
**Cut-offs**	1.1	11	0.17	39.69	46.98	8.2	33.28
**Sensitivity (%)**	76.9	76	61.5	64	61.5	61.5	69.2
**Specificity (%)**	73.7	78.4	51.4	75.7	52.6	73	70.3

Abbreviations: sCr—serum creatinine; SDMA—symmetric dimethylarginine; sCyst—serum cystatin C; uALPc—urinary alkaline phosphatase/creatinine ratio; uGGTc—urinary gamma-glutamyl transferase/creatinine ratio; uNAGc—urinary N-acetyl-β-D-glucosaminidase/creatinine ratio; uCystc—urinary cystatin C/creatinine ratio; AUC—area under the curve; CI—confidence interval; MMVD—myxomatous mitral valve disease.

**Table 6:** Correlations between the biomarkers studied in all dogs.

	sCr (mg/dL)	SDMA (μg/dL)	sCyst (mg/L)	uALPc (U/g)	uGGTc (U/g)	uNAGc (U/g)	uCystc (µg/g)	UPC	HGB (g/dL)
**sCr (mg/dL)**		0.478 *	0.350 **	0.066	−0.125	0.034	0.011	−0.013	−0.094
**SDMA (μg/dL)**	0.478 **		0.285 **	0.144	0.030	0.007	0.210	−0.007	−0.092
**sCyst (mg/L)**	0.350 **	0.285 **		0.193	0.016	0.014	0.189	0.191	−0.312 **
**uALPc (U/g)**	0.066	0.144	0.193		0.673 **	0.284 *	0.225 *	0.496 **	−0.041
**uGGTc (U/g)**	−0.125	0.030	0.016	0.673 **		0.312 **	0.068	0.546 **	−0.035
**uNAGc (U/g)**	0.034	0.007	0.014	0.284 *	0.312 **		0.305 **	0.084	−0.187
**uCystc (µg/g)**	0.011	0.210	0.189	0.225*	0.068	0.305 **		0.147	−0.290 **
**UPC**	−0.013	−0.007	0.191	0.496 **	0.546 **	0.084	0.147		0.004
**HGB (g/dL)**	−0.094	−0.092	−0.312 **	−0.041	−0.035	−0.187	−0.290 **	0.004	

Abbreviations: sCr—serum creatinine; SDMA—symmetric dimethylarginine; sCyst—serum cystatin C; uALPc—urinary ratio alkaline phosphatase/creatinine; uGGTc—urinary ratio gamma-glutamyl transferase/creatinine; uNAGc—urinary ratio N-acetyl B-D-glucosaminidase/creatinine; uCystc—urinary ratio cystatin C/creatinine; UPC—urinary ratio proteins/creatinine; HGB—haemoglobin concentration. * *p* < 0.05. ** *p* < 0.01.

## Data Availability

The original contributions presented in this study are included in the article/Supplementary Material. Further inquiries can be directed to the corresponding author.

## Supplementary Materials

The following supporting information can be downloaded at: https://www.mdpi.com/article/10.3390/vetsci13030243/s1, Table S1: Age-adjusted-ordered logistic regression models assessing the association between log-transformed creatinine-indexed urinary tubular biomarkers and ACVIM stage. Table S2: Absolute values of tubular biomarkers in healthy dogs and dogs with MMVD, overall and stratified by ACVIM stage.

## Abbreviations

The following abbreviations are used in this manuscript:

MMVD	Myxomatous mitral valve disease
ACVIM	American College of Veterinary Internal Medicine
CHF	Congestive heart failure
CvRD	Cardiovascular–renal axis disorders
CvRD_CH_	Cardiovascular–renal axis disorders secondary to chronic heart disease
SDMA	Symmetric dimethylarginine
sCr	Serum creatinine
sCyst	Serum cystatin C
UPC	Urinary ratio proteins/creatinine
uALPc	Urinary ratio alkaline phosphatase/creatinine
uGGTc	Urinary ratio gamma-glutamyl transferase/creatinine
uNAGc	Urinary ratio N-acetyl B-D-glucosaminidase/creatinine
uCystc	Urinary ratio cystatin C/creatinine
uNGALc	Urinary ratio neutrophil gelatinase-associated lipocalin/creatinine

## References

[1] Keene B.W., Atkins C.E., Bonagura J.D., Fox P.R., Häggström J., Fuentes V.L., Oyama M.A., Rush J.E., Stepien R., Uechi M. (2019). ACVIM Consensus Guidelines for the Diagnosis and Treatment of Myxomatous Mitral Valve Disease in Dogs. J. Vet. Intern. Med..

[2] Mattin M.J., Boswood A., Church D.B., McGreevy P.D., O’Neill D.G., Thomson P.C., Brodbelt D.C. (2015). Degenerative Mitral Valve Disease: Survival of Dogs Attending Primary-Care Practice in England. Prev. Vet. Med..

[3] Ljungvall I., Häggström J., Bussadori C. (2023). Myxomatous Valvular Disease. Textbook of Cardiovascular Medicine in Dogs and Cats.

[4] Pouchelon J.L., Atkins C.E., Bussadori C., Oyama M.A., Vaden S.L., Bonagura J.D., Chetboul V., Cowgill L.D., Elliot J., Francey T. (2015). Cardiovascular-Renal Axis Disorders in the Domestic Dog and Cat: A Veterinary Consensus Statement. J. Small Anim. Pract..

[5] Troia R., Sabetti M.C., Crosara S., Quintavalla C., Romito G., Mazzoldi C., Fidanzio F., Cescatti M., Bertazzolo W., Giunti M. (2022). Evaluation of Urinary Neutrophil Gelatinase-Associated Lipocalin to Detect Renal Tubular Damage in Dogs with Stable Myxomatous Mitral Valve Disease. J. Vet. Intern. Med..

[6] Jung H.B., Kang M.H., Park H.M. (2018). Evaluation of Serum Neutrophil Gelatinase–Associated Lipocalin as a Novel Biomarker of Cardiorenal Syndrome in Dogs. J. Vet. Diagn. Investig..

[7] Szczepankiewicz B., Paslawska U., Paslawski R., Gebarowski T., Zasada W., Michalek M., Noszczyk-Nowak A. (2019). The Urine Podocin/Creatinine Ratio as a Novel Biomarker of Cardiorenal Syndrome in Dogs Due to Degenerative Mitral Valve Disease. J. Physiol. Pharmacol..

[8] Choi B.S., Moon H.S., Seo S.H., Hyun C. (2017). Evaluation of Serum Cystatin-C and Symmetric Dimethylarginine Concentrations in Dogs with Heart Failure from Chronic Mitral Valvular Insufficiency. J. Vet. Med. Sci..

[9] Valente C., Guglielmini C., Baron Toaldo M., Romito G., Artusi C., Brugnolo L., Contiero B., Poser H. (2021). Plasmatic Dimethylarginines in Dogs With Myxomatous Mitral Valve Disease. Front. Vet. Sci..

[10] Orvalho J.S., Cowgill L.D. (2017). Cardiorenal Syndrome: Diagnosis and Management. Vet. Clin. N. Am. Small Anim. Pract..

[11] Cowgill L.D., Polzin D.J., Elliott J., Nabity M.B., Segev G., Grauer G.F., Brown S., Langston C., van Dongen A.M. (2016). Is Progressive Chronic Kidney Disease a Slow Acute Kidney Injury?. Vet. Clin. N. Am. Small Anim. Pract..

[12] International Renal Interest Society IRIS Guidelines. https://www.iris-kidney.com/iris-guidelines-1.

[13] Hokamp J.A., Nabity M.B. (2016). Renal Biomarkers in Domestic Species. Vet. Clin. Pathol..

[14] McKenna M., Pelligand L., Elliott J., Cotter D., Jepson R. (2020). Relationship between Serum Iohexol Clearance, Serum SDMA Concentration, and Serum Creatinine Concentration in Non-Azotemic Dogs. J. Vet. Intern. Med..

[15] Kim J., Lee C.M., Kim H.J. (2020). Biomarkers for Chronic Kidney Disease in Dogs: A Comparison Study. J. Vet. Med. Sci..

[16] Pereira A.F., Jota Baptista C., Faustino-Rocha A., Oliveira P.A., Coelho A.C. (2025). Renal Biomarkers in Companion Animals—A Review. Animals.

[17] Gembillo G., Visconti L., Giusti M.A., Siligato R., Gallo A., Santoro D., Mattina A. (2021). Cardiorenal Syndrome: New Pathways and Novel Biomarkers. Biomolecules.

[18] De Loor J., Daminet S., Smets P., Maddens B., Meyer E. (2013). Urinary Biomarkers for Acute Kidney Injury in Dogs. J. Vet. Intern. Med..

[19] Herget-Rosenthal S., Poppen D., Hüsing J., Marggraf G., Pietruck F., Jakob H.G., Philipp T., Kribben A. (2004). Prognostic Value of Tubular Proteinuria and Enzymuria in Nonoliguric Acute Tubular Necrosis. Clin. Chem..

[20] De Carvalho J.A.M., Piva S.J., Hausen B.S., Bochi G.V., Kaefer M., Coelho A.C., Duarte M.M.M.F., Moresco R.N. (2011). Assessment of Urinary γ-Glutamyltransferase and Alkaline Phosphatase for Diagnosis of Diabetic Nephropathy. Clin. Chim. Acta.

[21] Mahjoob M.P., Barzi F., Nassiri A., Kaveh A., Haghi M., Ghoddusi M., Sistanizad M. (2021). Adjunct Hypertonic Saline in Patients with Diffuse Edema Due to Heart Failure: A Randomized Double-Blinded Clinical Trial. Iran. J. Pharm. Res..

[22] Damman K., Van Veldhuisen D.J., Navis G., Vaidya V.S., Smilde T.D.J., Westenbrink B.D., Bonventre J.V., Voors A.A., Hillege H.L. (2010). Tubular Damage in Chronic Systolic Heart Failure Is Associated with Reduced Survival Independent of Glomerular Filtration Rate. Heart.

[23] Zhao T., Chen G., Zhu S., Zhao C., Jin C., Xie Y., Xiang M. (2023). Prognostic Value of Urinary N-Acetyl-β-d-Glucosaminidase as a Marker of Tubular Damage in Patients with Heart Failure and Mitral Regurgitation. Rev. Cardiovasc. Med..

[24] Acierno M.J., Brown S., Coleman A.E., Jepson R.E., Papich M., Stepien R.L., Syme H.M. (2018). ACVIM Consensus Statement: Guidelines for the Identification, Evaluation, and Management of Systemic Hypertension in Dogs and Cats. J. Vet. Intern. Med..

[25] Rishniw M. (2018). Murmur Grading in Humans and Animals: Past and Present. J. Vet. Cardiol..

[26] Almy F.S., Christopher M.M., King D.P., Brown S.A. (2002). Evaluation of Cystatin C as an Endogenous Marker of Glomerular Filtration Rate in Dogs. J. Vet. Intern. Med..

[27] Muñoz J., Soblechero P., Duque F.J., Macías-García B., Ruiz P., Zaragoza C., Barrera R. (2017). Effects of Oral Prednisone Administration on Serum Cystatin C in Dogs. J. Vet. Intern. Med..

[28] Ilchyshyn N.P., Villiers E., Monti P. (2019). Validation of a spectrophotometric method for GGT measurement in canine urine and determination of the urine GGT-to-creatinine ratio reference interval and biological variation in 41 healthy dogs. J. Vet. Diagn. Investig..

[29] Ruiz P., Durán Á., Duque F.J., González M.A., Cristóbal J.I., Nicolás P., Pérez-Merino E.M., Macías-García B., Barrera R. (2023). Urinary Cystatin C and N-Acetyl-Beta-D-Glucosaminidase (NAG) as Early Biomarkers for Renal Disease in Dogs with Leishmaniosis. Vet. Parasitol..

[30] Nabity M.B., Lees G.E., Cianciolo R., Boggess M.M., Steiner J.M., Suchodolski J.S. (2012). Urinary biomarkers of renal disease in dogs with X-linked hereditary nephropathy. J. Vet. Intern. Med..

[31] Ronco C., Haapio M., House A.A., Anavekar N., Bellomo R. (2008). Cardiorenal Syndrome. J. Am. Coll. Cardiol..

[32] Duque J., Barrera R., Caro A., Daza M.Á., Marcos G., Mogollón M.V., Ghipayo D., Casamian D., Martínez F. (2019). Cardiovascular–Renal Axis Disorders in Dogs and Cats.

[33] Nicolle A.P., Chetboul V., Allerheiligen T., Pouchelon J.-L., Gouni V., Tessier-Vetzel D., Sampedrano C.C., Lefebvre H.P. (2007). Azotemia and Glomerular Filtration Rate in Dogs with Chronic Valvular Disease. J. Vet. Intern. Med..

[34] Martinelli E., Locatelli C., Bassis S., Crosara S., Paltrinieri S., Scarpa P., Spalla I., Zanaboni A.M., Quintavalla C., Brambilla P. (2016). Preliminary Investigation of Cardiovascular–Renal Disorders in Dogs with Chronic Mitral Valve Disease. J. Vet. Intern. Med..

[35] Yun H., Koo Y., Yun T., Chae Y., Lee D., Cha S., Kim J., Kim H., Pyo Yang M., Teck Kang B. (2023). Evaluation of Progression of Chronic Kidney Disease in Dogs with Myxomatous Mitral Valve Disease. Front. Vet. Sci..

[36] Iwasa N., Kumazawa R., Shimizu M., Okamoto T., Kawabe M., Iwata M., Watanabe K., Kobatake Y., Takashima S., Nishii N. (2025). Prognostic Value of Circulating Cardiac and Renal Biomarkers in Dogs with Myxomatous Mitral Valve Disease. Res. Vet. Sci..

[37] Iwasa N., Kumazawa R., Nomura S., Shimizu M., Iwata M., Hara M., Kawabe M., Kobatake Y., Takashima S., Nishii N. (2023). Prognostic Value of Serum Cystatin C Concentration in Dogs with Myxomatous Mitral Valve Disease. J. Vet. Intern. Med..

[38] Giorgi M.E., Mochel J.P., Yuan L., Adin D.B., Ward J.L. (2022). Retrospective Evaluation of Risk Factors for Development of Kidney Injury after Parenteral Furosemide Treatment of Left-Sided Congestive Heart Failure in Dogs. J. Vet. Intern. Med..

[39] Sabetti M.C., Fasoli S., Crosara S., Quintavalla C., Romito G., Troìa R., Fidanzio F., Mazzoldi C., Monari E., Dondi F. (2025). Neutrophil Gelatinase-Associated Lipocalin (NGAL) as a Biomarker of Acute Kidney Injury (AKI) in Dogs with Congestive Heart Failure (CHF) Due to Myxomatous Mitral Valve Disease (MMVD). Animals.

[40] Guglielmini C., Valentini C.M., Contiero B., Valente C., Poser H. (2021). Red Cell Distribution Width Has a Negative Prognostic Role in Dogs with Myxomatous Mitral Valve Disease. Animals.

[41] McCullough P.A. (2021). Anemia of Cardiorenal Syndrome. Kidney Int. Suppl..

[42] Polzin D.J. (2011). Chronic Kidney Disease in Small Animals. Vet. Clin. N. Am. Small Anim. Pract..

[43] Yu I.B.Y., Huang H.P. (2016). Prevalence and Prognosis of Anemia in Dogs with Degenerative Mitral Valve Disease. Biomed. Res. Int..

[44] Kumiega E., Kobak A.K., Noszczyk-Nowak A., Kasztura M. (2024). Iron Parameters Analysis in Dogs with Myxomatous Mitral Valve Disease. BMC Vet. Res..

[45] Jung M.-J., Kim J.-H. (2023). Prognostic Efficacy of Complete Blood Count Indices for Assessing the Presence and the Progression of Myxomatous Mitral Valve Disease in Dogs. Animals.

[46] Kocaturk M., Saril A., Oz A.D., Rubio C.P., Ceron J.J., Yilmaz Z. (2024). Neutrophil-to-Lymphocyte Ratio and Red Blood Cell Distribution Width to Platelet Ratio and Their Relationships with Inflammatory and Antioxidant Status in Dogs with Different Stages of Heart Failure Due to Myxomatous Mitral Valve Disease. Vet. Res. Commun..

[47] Rubio C.P., Saril A., Kocaturk M., Tanaka R., Koch J., Ceron J.J., Yilmaz Z. (2020). Changes of Inflammatory and Oxidative Stress Biomarkers in Dogs with Different Stages of Heart Failure. BMC Vet. Res..

[48] Miyagawa Y., Takemura N., Hirose H. (2010). Assessments of Factors That Affect Glomerular Filtration Rate and Indirect Markers of Renal Function in Dogs and Cats. J. Vet. Med. Sci..

[49] Valente C., Guglielmini C., Domenech O., Contiero B., Zini E., Poser H. (2020). Symmetric Dimethylarginine in Dogs with Myxomatous Mitral Valve Disease at Various Stages of Disease Severity. PLoS ONE.

[50] O’Neill D.G., Elliott J., Church D.B., Mcgreevy P.D., Thomson P.C., Brodbelt D.C. (2013). Chronic Kidney Disease in Dogs in UK Veterinary Practices: Prevalence, Risk Factors, and Survival. J. Vet. Intern. Med..

[51] Marynissen S.J.J., Willems A.L., Paepe D., Smets P.M.Y., Picavet P., Duchateau L., Daminet S. (2017). Proteinuria in Apparently Healthy Elderly Dogs: Persistency and Comparison Between Free Catch and Cystocentesis Urine. J. Vet. Intern. Med..

[52] Adin D., Atkins C., Papich M.G. (2018). Pharmacodynamic Assessment of Diuretic Efficacy and Braking in a Furosemide Continuous Infusion Model. J. Vet. Cardiol..

[53] Sabetti M.C., Fidanzio F., Troìa R., Perissinotto L., Romito G., Mazzoldi C., Quintavalla C., Crosara S., Dondi F. (2022). Effect of Sampling Time on Urinary Electrolytes Following Oral Furosemide Administration in Dogs with Myxomatous Mitral Valve Disease. J. Vet. Cardiol..

[54] Nabity M., Hokamp J. (2023). Urinary Biomarkers of Kidney Disease in Dogs and Cats. Vet. Clin. N. Am. Small Anim. Pract..

[55] Clemo F.A.S. (1998). Urinary Enzyme Evaluation of Nephrotoxicity in the Dog. Toxicol. Pathol..

[56] Bosomworth M.P., Aparicio S.R., Hay A.W.M. (1999). Urine N-Acetyl-Beta-D-Glucosaminidase—A Marker of Tubular Damage?. Nephrol. Dial. Transplant..

[57] Grauer G.F. (1996). Prevention of Acute Renal Failure. Vet. Clin. N. Am. Small Anim. Pract..

[58] Jepson R.E., Vallance C., Syme H.M., Elliott J. (2010). Assessment of Urinary N-Acetyl-β-D-Glucosaminidase Activity in Geriatric Cats with Variable Plasma Creatinine Concentrations with and without Azotemia. Am. J. Vet. Res..

[59] Heiene R., Moe L., Mølmen G. (2001). Calculation of Urinary Enzyme Excretion, with Renal Structure and Function in Dogs with Pyometra. Res. Vet. Sci..

[60] Ruiz P., Sevidane I., Durán A., García A.B., Macías-García B., Barrera R. (2025). Urinary Gamma-Glutamyl Transferase as an Early Biomarker of Renal Disease in Dogs with Leishmaniosis. Vet. Sci..

[61] Nivy R., Avital Y., Aroch I., Segev G. (2017). Utility of Urinary Alkaline Phosphatase and γ-Glutamyl Transpeptidase in Diagnosing Acute Kidney Injury in Dogs. Vet. J..

[62] Smets P.M.Y., Meyer E., Maddens B.E.J., Duchateau L., Daminet S. (2010). Urinary Markers in Healthy Young and Aged Dogs and Dogs with Chronic Kidney Disease. J. Vet. Intern. Med..

[63] Paltrinieri S., Mangiagalli G., Ibba F. (2018). Use of Urinary γ-Glutamyl Transferase (GGT) to Monitor the Pattern of Proteinuria in Dogs with Leishmaniasis Treated with N-Methylglucamine Antimoniate. Res. Vet. Sci..

[64] D’Amico G., Bazzi C. (2003). Urinary Protein and Enzyme Excretion as Markers of Tubular Damage. Curr. Opin. Nephrol. Hypertens..

[65] Vaidya V.S., Ferguson M.A., Bonventre J.V. (2008). Biomarkers of Acute Kidney Injury. Annu. Rev. Pharmacol. Toxicol..

[66] Monti P., Benchekroun G., Berlato D., Archer J. (2012). Initial Evaluation of Canine Urinary Cystatin C as a Marker of Renal Tubular Function. J. Small Anim. Pract..

[67] Selin A.K., Lilliehöök I., Strage E.M., Larsson A., Pelander L. (2025). Urinary Cystatin C, Glucose, Urea, and Electrolytes in Dogs at Various Stages of Chronic Kidney Disease. J. Vet. Intern. Med..

[68] Akama Y., Matsue Y., Maeda D., Dotare T., Sunayama T., Iso T., Fujimoto Y., Nakade T., Yatsu S., Ishiwata S. (2025). Prognostic Values of Proteinuria in Patients with Acute Heart Failure. J. Cardiol..

[69] Rangaswami J., Bhalla V., Blair J.E.A., Chang T.I., Costa S., Lentine K.L., Lerma E.V., Mezue K., Molitch M., Mullens W. (2019). Cardiorenal Syndrome: Classification, Pathophysiology, Diagnosis, and Treatment Strategies: A Scientific Statement from the American Heart Association. Circulation.

[70] Crosara S., Fidanzio F., Oricco S., Dondi F., Mazzoldi C., Monari E., Romito G., Sabetti M.C., Troìa R., Quintavalla C. (2024). Association between Echocardiographic Indexes and Urinary Neutrophil Gelatinase-Associated Lipocalin (UNGAL) in Dogs with Myxomatous Mitral Valve Disease. Res. Vet. Sci..

[71] Petit A.M., Gouni V., Tissier R., Trehiou-Sechi E., Misbach C., Pouchelon J.L., Lefebvre H.P., Chetboul V. (2013). Systolic Arterial Blood Pressure in Small-Breed Dogs with Degenerative Mitral Valve Disease: A Prospective Study of 103 Cases (2007–2012). Vet. J..

[72] Damman K., Ng Kam Chuen M.J., MacFadyen R.J., Lip G.Y.H., Gaze D., Collinson P.O., Hillege H.L., Van Oeveren W., Voors A.A., Van Veldhuisen D.J. (2011). Volume Status and Diuretic Therapy in Systolic Heart Failure and the Detection of Early Abnormalities in Renal and Tubular Function. J. Am. Coll. Cardiol..

