# Supplementary material for: Evaluation of Urinary Tubular Biomarkers in Dogs with Myxomatous Mitral Valve Disease Across ACVIM Stages
Source: Vet Sci. 2026 Mar 3;13(3):243. doi: 10.3390/vetsci13030243 (PMC13030666; doi:10.3390/vetsci13030243)
Supplement: Supplementary file 1 [file vetsci-13-00243-s001.zip › Supplementary Material Table S1.pdf]

Table S1. Age-adjusted-ordered logistic regression models assessing the association between log-transformed creatinine-indexed urinary tubular biomarkers and ACVIM stage.

**Biomarker**	**OR**	**95% CI**	**p-value**
uALPc*	1.87	1.35–2.61	<0.001
uGGTc*	3.04	1.57–5.88	0.001
uNAGc*	1.30	1.07–1.59	0.009
uCystc*	1.71	1.06–2.77	0.028

Abbreviations: uALP. urinary alkaline phosphatase; uGGT. urinary gamma-glutamyl transferase; uCyst. urinary cystatin C; uNAG. urinary N-acetyl B-D-glucosaminidase; OR. odds ratio; CI. confidence interval.

*Log-transformed variables were used due to the skewness and wide dispersion typical of urinary enzymatic markers.
