# Supplementary material for: Evaluation of Urinary Tubular Biomarkers in Dogs with Myxomatous Mitral Valve Disease Across ACVIM Stages
Source: Vet Sci. 2026 Mar 3;13(3):243. doi: 10.3390/vetsci13030243 (PMC13030666; doi:10.3390/vetsci13030243)
Supplement: Supplementary file 1 [file vetsci-13-00243-s001.zip › Supplementary Material Table S2.pdf]

Table S2. Absolute values of tubular biomarkers in healthy dogs and dogs with MMVD, overall and stratified by ACVIM stage.

	**Control Group**	**MMVD Group**	**B1 Group**	**B2 Group**	**C+D Group**
**uALP (U/L)**	7.5 (1-12.5) ^*&π	22 (6-63.5) ^	24.5 (9.5-46.5) *	14 (9-62) &	21.5 (6-83) π
**uGGT (U/L)**	37.5 (26.5-56.5)	46.5 (20.75-83)	52 (42-84)	60 (31-127) -	27.5 (9-58) -
**uCyst (mg/L)**	0.03 (0.012-0.05)	0.025 (0.01-0.05)	0.03 (0.01-0.07)	0,02 (0,015-0.05)	0.02 (0.01-0-04)
**uNAG (U/L)**	2.95 (0,1-7.44)	4.61 (0.1-8.14)	3.58 (0.1-7.4)	7.43 (1.47-13.28)	4.24 (2.27-8.52)

Abbreviations: uALP. urinary alkaline phosphatase; uGGT. urinary gamma-glutamyl transferase; uCyst. urinary cystatin C; uNAG. urinary N-acetyl B-D-glucosaminidase. Values are presented as median (IQR). *$p<0.05$ indicates a statistically significant difference between control group and B1 group; & between control group and B2 group; π between control group and C+D group; - between B2 group and C+D group; ^ between control group and MMVD group.
